# Climate change induced a progressive shift of livelihood from cereal towards *Khat (Chata edulis)* production in eastern Ethiopia

**DOI:** 10.1016/j.heliyon.2022.e12790

**Published:** 2023-01-03

**Authors:** Daniel Assefa Tofu, Kebede Wolka

**Affiliations:** a School of Natural Resource, Ambo University, Ethiopia; b Wondo Genet College of Forestry & Natural Resource, Hawassa University, Ethiopia

**Keywords:** Climate change, *Khat*, Natural resource rehabilitation, Water scarcity, Food security

## Abstract

Global climate change affects smallholders in sub-Saharan Africa. To cope with the changing situation, farmers employ adaptation strategies such as adjusting their livelihoods. The objective of this research was to evaluate the effects of climate change on the gradual transition in livelihood from cereal production to *Khat* plantation in west Harareghe, Ethiopia. Data was gathered through interviews with 273 households, 8, focus groups, and 12 key informants. Descriptive statistics and an econometric model were used to analyze quantitative data, whereas content analysis with step preprocesses was utilized to analyze and report qualitative data. . The farm households responded that climatic extremes are posing severe impacts on soil fertility (80%), crop production (93%), livestock production (91%), water scarcity (92%), and adaptive capacity of the farmers (81%). As a result, about 86% of the farmers' have been putting more emphasis and progressively shifting their livelihood from cereal to *Khat (Chata edulis)* crop plantation. Asides from the shrinking livelihood due to climatic shocks, high market access with better price and profitability, drought and water scarcity tolerance, ability to grow in degraded land, suitability for intercropping and three to five times harvest in a year are mentioned as the blessings attracted farmers’ to shift to *Khat* production. To reduce susceptibility to climate change impacts, approximately 81%, 78%, and 77% of framers, respectively, used managerial (e.g., intercropping and petty trade), technological (e.g., terracing and improved crop variety), and policy-driven (i.e., productive safety net program) strategies. In addition to this, agro-ecology, yield reduction, wealth, perceived on set of rain, soil infertility, access to markets and credit, institutional participation, land size, dependency ratio, irrigation, and access to early warning systems were found to significantly influence the adoption decision of farmers in the study area. Therefore, policy makers and planners are advised to design techniques to manage climate-induced extreme episodes and produce area specific strategies capable to increase the productivity of cereal crop and livestock.

## 1 Introduction

Climate change is becoming a global issue, affecting more people in developing parts of the world. Among the economic sectors, agriculture, such as crop yields, is highly affected by the changes in rainfall and temperature below or above a threshold [1]. Both the level of temperature and the amount and timing of rainfall are the major factors of crop production, which determine the growth and quantity of harvest. In agriculture, it is not only amount of rainfall that matters for crop production but also level of variability from the mean rainfall affects production. When the rainfall diverges from the mean amount (both upward and downward), the level of production has significantly diminished for all crop types [2]. Besides, the increase in temperature has huge implications for the growth and productivity of crops through its negative effects on soil moisture, potential evapotranspiration, and heat stress on crops [3].

The global effects of high temperatures, the boundary and suitable areas for cultivation have been changing. The change in the production climate might be a crisis for certain areas but might be an opportunity for others [4]. There is a consensus among scholars that crop production will be steadily and negatively affected by climate change in the future in low-latitude countries, while it has a high probability of having a positive effect in northern latitudes [5]. In general, evidence of observed changes in climate extremes such as heat waves, variability in rainfall, droughts, and tropical cyclones, and, in particular, their effects on every inhabited region across the globe with a significant impact on agriculture, has strengthened across the globe [6]. Despite the fact that the impacts of climate change and variability are *trans*-boundary; they have started to increase the spatial concentration of crop production in some corners and are exacerbating the relative differences in crop output in other locations [7].

Weather and climate variability are believed to be the prominent determinants of agricultural production systems, and the recent global trends in the change of climate variables are assumed as to be responsible for substantial effects on crop yield trends [8]. This is due to the fact that climate variability and change have many dimensions of effects, particularly on agriculture, by influencing the emergence and distribution of crop pests, weeds, and crop parasites; increasing the occurrence and frequency of extreme weather events; reducing water supplies available for irrigation; and increasing the severity of soil erosion [9]. Due to an upward rise in temperature, in Sub-Saharan Africa, the growing period will reduce, and the planting date also needs to change accordingly [10,11]. Moreover, climate change and variability are proven to decrease the crop rotation period, so farmers need to consider crop varieties, sowing dates, crop densities, and fertilization levels when planting crops [12]. In general, in this century, global agriculture and the people dependent on it have been hardily affected by climate change [13].

Climate change, in general, has a particularly negative influence in locations where rain-fed agriculture is practiced [14]. Smallholder farmers, in particular, depend on rain-fed produce with limited resources and assets, making rural livelihoods precarious and human welfare vulnerable to changing weather patterns [15,16]. Increased frequency and intensity of weather-related extremes, such as droughts, floods, and heat waves, as well as gradual temperature changes, will have worsening consequences on smallholder farmers' livelihoods [17] (DFID, 2013). Smallholder farmers must use new production and consumption techniques and technologies to either mitigate the effects of catastrophic occurrences or adapt to an already altered climate. The need for water management as well as the quest for cultivars that can be harvested quickly or endure water stress for efficient adaptation is demonstrated by the unpredictability of rainfall distribution [18]. Agriculture could adapt to more extreme events by using new tactics such as cultivating more tolerant crops and changing management practices [2].

High levels of vulnerability to climate change and insufficient adaptive capability have been linked to financial and institutional issues in developing countries, particularly in Africa [19]. Climate change, on the other hand, has the potential to cause a slew of issues, creating a vicious spiral. When climate change affects crop yields, for example, it has a direct impact on farmers' income, which in turn has an impact on their ability to adapt to new technology [20]. Climate-related shocks, along with low technology and high poverty, leave individuals with few options or resources to adapt, particularly in developing countries [1]. In an effort to overcome climate change and associated problems, communities sometimes autonomously shift their livelihood from the one they experienced for a long time to a new way of farming. Farmers less likely change their habitual agricultural practices to cope with the climate in East Wollega [21]. But, farmers in arid and semi-arid regions are intensifying and changing from rangeland to mixed systems; transitioning from pastoralism to agro-pastoralism due to a decline in rangeland; and thereby deciding to follow intensive types of production systems to live with an already changed climate [19,22].

As an adaptation strategy, farmers in West Harareghe, Ethiopia are expanding the plantation of *Khat* (*Chata edulis*) instead of other cereal crops. *Khat* crop is found primarily in Ethiopia, Kenya, Somalia, Sudan, Madagascar, Yemen, and South Africa, but it is also found on smaller-scale in Turkistan and Afghanistan [23,24]. In Ethiopia, over two million small-scale farmers produce *Khat* on more than 250,000 ha of land, mainly to diversify their income and ensure food security [25,26]. Accordingly, *Khat* production has been growing steadily in Ethiopia and the country is ranked as the leading producer of *Khat* globally and exports it to neighboring countries, e.g., Djibouti and Somalia [27]. It is now one of Ethiopia's largest sources of internal tax revenue and one of the fastest growing export commodities, with annual earnings in the hundreds of millions of dollars [28,29]. For instance, excluding the number of tax collections at the regional, zonal, and district levels, the national government collected an estimated $289 million in 2010 from *Khat* [30]. Increased spatial disparities in crop production and its output force populations to seek and shape livelihood opportunities [7].

Apart from its national economic advantages, *Khat* crop has diversity of positive rewards for to subsistence farmers who plant it. Among these, its genetic ability to tolerate climatic extremes, particularly drought conditions, for several months is the most mentioned one [31]. Another advantage of *Khat* is its ability to grow in a wide range of ago-ecological conditions and to withstand climatic extremes. It can also be harvested more than three times each year, providing a consistent source of income for farm households [31]. Furthermore, the highest return on the investment costs relative to other grain crops [32–34], its ability to provide a strong and consistent source of revenue, which helps to secure livelihood needs of the households throughout the year [34,35], and its lesser risky to grow than grains and coffee, particularly in the face of pressing climate variability-tempted shocks [36], *Khat* can be grown on a small plot of land [32,37]. Furthermore, its better financial reward for households, limited land holdings, steady market circumstances, and a reduction in government subsidies to buy fertilizer for annual crops are the blessings that attract the attention of farmers to grow *Khat* instead of cereal crops [38].

There is a substantial amounts of literature available on various aspects of the *Khat* crop, including expansion in the Ethiopian highlands [36]; on its socio-economic advantage [31]; on its benefit to climate change adaptation [39]; on its challenge [3]; on its psychological, economic, and social impact on the users [40]; on its benefit in reducing farming household vulnerability to climate variability [35]; on its implication on land use practice [41]; on its management practice [42]; on its suitability to practice agroforestry [38]; and on its socio-economic advantage to farmers [24,33,43].

However, considering its livelihood advantage to smallholder farmers in the pace of climate change and the factors that pull farmers to extensively plant *Khat* crops instead of cereal crops; it was not studied in a comprehensive manner. The goal of this study was to fill in the knowledge gaps about the extent of climatic variability and change impacts on cereal rope production as well as subsistence farmers’ livelihoods until pushing farmers from the most experienced farming systems to look into other types of crops, which were not commonly practiced as the main foodstuff crop previously, to adopt the severity of climate variability and change-induced extreme episodes. Therefore, this study was initiated to answer the following questions: (I) What is the perception of climate change, its causes, and local indicators? (II) What are the implications of climate change for diverse livelihoods? (III) How has climate change affected livelihoods in the West Harareghe zone? (IV) What is the economic and livelihood value of the *Khat* plant that encourages farmers to plant *Khat* rather than food crops? (V) What adaptation strategies do farmers use and their determinants to live with the already changed impacts of climate change-induced shocks?

## 2 Materials and method

### 2.1 Study area description

**Fig. 1:**
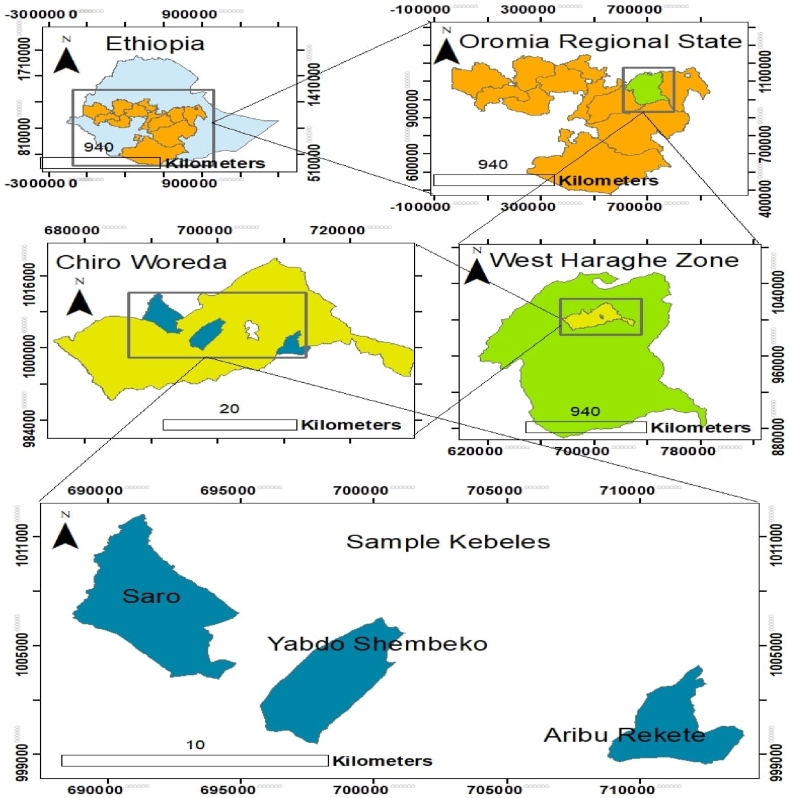
Map of the study area.

The study was conducted in the Chiro district, located in the West Hararghe zone of the Oromia Regional State of Ethiopia. The area's coordinates are 9°5′–9°6′N latitude and 40°15′–40°54’′E longitude (Fig. 1). The district is 326 km away from Addis Ababa in the east direction. The elevation ranges from 1500 to 2500 m above sea level, with 56.4% lowland (33 kebeles), 33.3% mid-altitude (13 kebeles), and 10.3% (4 kebeles) highland [44]. In the lowlands and highlands, the annual average temperature ranges from 27.5 °C to 38.5 °C, with annual rainfall ranging from 600 mm to 1000 mm in the lowlands and highlands, respectively [45]. Rainfall is bimodal and erratic, with the main rainy season occurring from June to September in the highlands and midlands and form March to April for the lowland. The short rainy season runs from March to May for the highland and midland. The district is mainly characterized by steep slopes and rugged topography, which makes it highly vulnerable to erosion and soil degradation [46]. Subsistence agriculture is a major livelihood in the area, and annual crops such as maize, barley, and sorghum are commonly cultivated crops.

*Khat* is a cash crop in this area and is grown in places not too far from the main road in order to easily supply to major markets as it is a perishable commodity [47]. There has been no agreement among scholars regarding the origin of the *Khat* crop in the world. Numan (2012) noted that the origin of *Khat* is around the southern Red Sea, Ethiopia, or Yemen. There is also literature that believes that it is native to Ethiopia [48]. For the first time, it was found in Yemen between the 1st and 6th centuries, and later on, the Danish botanist and physician Forsskal gave the name *Catha edulis* to the plant growing in the mountains of Yemen in 1762 [49,50]. However, there is no definite evidence for specific time of introduction and the origin of *Khat* either *in* Yemen or Ethiopia [34]. Currently, Ethiopia is the leading *Khat* producer in Africa and worldwide. The Hararghe area, where this study was conducted, takes a high share. The *Khat* production area is expanding. About 248,000 ha were cultivated in 2014–15 [51]. Over the last 15 years, the amount of land devoted to *Khat* has increased by 160% [27]. From just a few thousand hectares in the 1950s, *Khat* is now grown on one-quarter million hectares of land in Ethiopia by millions of farmers, particularly in the eastern parts of the country (both West and East Hararghe) considering its diverse advantages including better income and drought episode resistance [27,29]. The demand for the plant is not only the domestic market, but it also traded and consumed mainly in Somalia, and Djibouti, and in some parts of Europe and North America, even if the plant seen as a drug of abuse in those countries [52]. *Khat* is becoming among the leading exportable agricultural commodities and it covers 4% of the country's export earnings and shares 9.4% of total merchandise export [53].

### 2.2 Methodology

#### 2.2.1 Sampling and data collection

The Chiro district in the West Harareghe zone was purposefully selected. The reasons for using purposive selection were to investigate the severity of climate variability and climate change-induced episodes, their impact on the food production and livelihood systems of the farmers’, and their adaptation strategies against the severity of impacts. From Chiro district, three kebeles (the smallest level of government administrative units in Ethiopia), namely, Yabdo Shemebeqo, Saro, and Arbu Rekete, were randomly selected. A total of 273 rural households (both male-headed and female-headed) were randomly selected from those kebeles for individual interviews (N = 10,658 using n = N/1 + N(e^2^) [54]. The head of the household was allowed to respond to the questionnaire. Like many parts of Ethiopia, where the male is considered the head of the household, he responded to the questionnaire. The female-headed household, which is due to divorce and a widow, responded to the questionnaire. In the absence of the husband during fieldwork, the wife responded to the questionnaire. Local experts such as agricultural development agents conducted major proportion of the questionnaire in 2021 after getting practical training and guidance from the lead researcher. The questionnaire was written in English but the data collector asked in the local language (Afaan Oromoo). Furthermore, focus group discussions (FGDs) were conducted with specifically selected community members. Experts in agriculture and natural resource development, the environment and climate change, water resource development, and leaders of the kebeles were also interviewed as key informants (KII).

A mixed approach to the research, quantitative and qualitative, along with the phenomenological design, was used. Quantitative data was collected from individual households using a questionnaire. It is acknowledged that whilst validity and reliability are predominantly derived from quantitative research, qualitative studies provide meaningful in-depth insights through subjective interpretations of experiences that provide plausible answers in relation to social phenomena [55]. A mixed methods approach promotes triangulation and rigor [56,57]. A phenomenological design was used to explore people's day-to-day life experiences connected to their strong strive to secure livelihoods [58], which is applied in qualitative research because it attempts to understand how participants make sense of their experiences [59].

FGD, KII, and field observation were used to collect qualitative data, while the household survey method was used to collect quantitative data. To collect quantitative data, a face-to-face interview was conducted with the head (i.e., either male or female) of farm households using a structured questionnaire. The questionnaire focuses on major livelihoods, climate change and their indicators, causes of climate change, climate change effects and adaptation strategies, and livelihood shifts to overcome climate change. The survey data were collected by trained experts working in the district with a bachelor's degree in crop, animal, and other sciences, using the local language, Afaan Oromoo. Aged farmers, who were not included in the survey, were chosen for FGD discussions. Older farmers were selected with particular attention not to focus on the short period of experience of the study area because the issues of climate change and its variability as well as *Khat* plantation are better understood by older farmers. However, the views of innovative and risk-averse youth segments of the community were also captured by household survey methods. For this purpose, elderly women and men of 8–12 individuals were set in groups, and the discussions were managed by researchers using a discussion guiding open-ended questions to brainstorm on issues of livelihood, climate change, and adaptation methods. Accordingly, a total of eight FGDs, four from women's and men's groups, and 12 KIIs were held across the study area. During the discussion period, a moderator tried to create a room where all members of the group had an equal chance to express their feelings and encouraged them to have a natural discussion. Besides, field observations were also given attention for the purpose of triangulating what was said by the people involved in the survey, FGD, and KII.

#### 2.2.2 Data analysis

Quantitative data was analyzed using Statistical Packages for Social Science (SPSS) version 20 and STATA/IC 14 computer software. Farmers’ responses were summarized descriptively. Qualitative data was analyzed following step-by-step procedures. Voice data that was gathered both from FGD and KII was first transcribed and transformed into text forms of data. After reading and re-reading the text (transcribed data), the patterns of ideas and concepts were identified and organized into coherent categories that allowed for a summary of the entire data set. In qualitative data analysis, categorization is a principal step of analysis in the pursuit of meaning for different words and phrases through bringing together a number of observations, ideas, and concepts that we consider similar in some respects, by implied contrast with other ideas, concepts, or observations [59]. Content analysis was used to produce parts of this report in corroboration with the quantitative data.

An economic model was used in order to identify the determinants of adaptation strategies by farmers. Regarding this, to analyze the determinants of adoption decisions by farmers, two choices of econometric models, including Multinomial Probit (MNP) and Multinomial Logit (MNL), were used by various scholars. Concerning the theoretical and formulation, both models is similar, but MNL is preferred because its cumulative distribution function (CDF) is logistic whereas that of MNP is a normal distribution. According to Ref. [60], the MNP model also does not enable precision robustness as it fails to allow the researcher to adjust for covariates, as in the case of MNL. Hence, in this study, the MNL model was chosen to determine factors influencing farmers’ adoption of climate change adaptation strategies over the MNP. In addition to this, MNL has one more advantage; that is, it permits the analysis of decisions across more than two categories, allowing the determination of selection probabilities for different categories [61,62].

To define the MNL model, let *y* denote a random variable taking on the values {1, 2, ….,*J}* for *J,* a positive integer, and let x denote a set of conditioning variables. In this case, *y* denotes adaptation options or categories, and x contains different socio-economic, institutional, and environmental variables (discussed in part 3.8). The quest is how the changes in the elements of x (explanatory or independent variable) affect the response probabilities for *j = 2, …., J*.

Assume x be a 1 x k vector with first elementary unit. The MNL model has response probabilities and its functional form is specified in Equation (1) as [60]:

1 $$P\left(y=j/x\right)=\frac{\exp\left(x\beta j\right)}{[1+\sum_{h=1}^{j}\exp\left(x\beta h\right),j=1,.\;.\;.\;.\;.J}$$

where $\beta_{j}$ is k x 1, j = 1, …., J. In this study, the adaptation strategies that farmers have been using at different level to against the impacts of climate change-induced shocks are collected individually and trimmed under four categories such as technological, behavioral, managerial, and policy driven.

Unbiased and consistent parameter estimates of the MNL model in Eq. (1) require the assumption of independence of irrelevant alternatives (IIA) to hold. In particular, the IIA assumption requires that the probability of using a certain adaptation method by a given household needs to be independent from the probability of choosing another adaptation method (i.e., Pj/Pk) is independent of the remaining probabilities. The premise of the IIA assumption is that there are independent and homoscedastic disturbance terms in the basic model in Eq. (1). The parameter estimates of the MNL model provide only the direction of the effect of the explanatory variables on the response variable, but estimates do not represent either the actual magnitude of change or probabilities [62,63,64,65]. Therefore, the formula or equation that enables us to measure the marginal probabilities of the expected change in probability of a particular choice being made concerning a unit change in an independent variable from the mean is demanding. Accordingly, differentiating Equation (1) concerning the explanatory variables that provide marginal effects of the explanatory variables as follow:

2 $$\frac{\partial Pj}{\partial Xk}=Pj\;\left(\beta jk-\sum_{J=1}^{J-1}Pj\beta jk\right)$$

The marginal effects, or marginal probabilities, are functions of the probability itself and measure the expected change in probability of a particular choice being made with respect to a unit change in an independent variable from the mean [66]. Finally, the model that tested for the validity of the IIA assumptions by using Hausman's test failed to reject the null hypothesis of independence of the climate change adaptation strategies, implying that the MNL specification is appropriate to model climate change-induced shock adaptation practices of subsistence farmers in the study area. In addition to this, multicollinearity problem were checked before running the model further. To this effect, all the fifteen explanatory variables were checked for multicollinearity using the Variance Inflation Factor (VIF). The average VIF for all variables was 1.66 (ranging from 1.03 to 2.43), suggesting that multicollineraity is not a problem in model estimation because it is below the threshold, 10.

## 3 Results and discussion

### 3.1 Socio-economic and livelihood characteristics of the study area

In the study area, 82% of the respondents were male-headed households, while 18% were interviewed from the female-headed households. Concerning the educational background of the respondents, the majority (59%) were able to read and write, whereas the rest (about 41%) were not able to read and write. The average age of the respondents were 47 with minimum 21 and maximum 75 years old. In the study area, the average family size is six, with range of four to nine. The household owns 1–3.3 ha (average 1.59 ha), which is higher than the national average rural land holding (1 ha). However, it was not only the land that was assigned to cultivation but rather shows the entire land owned by the households, including settlement, grazing, and the area covered by trees.

Crop-livestock mixed agriculture is the most common source of income and source of livelihood for households across the study areas. According to the household respondents, maize, sorghum, and teff arecommon crops grown in the area, and they provide a substantial source of income for around 40% of households. *Khat* is also a common cash crop farmed by local farmers, contributing to smallholder farmers' income. About 43% of farmers believe that *Khat* is their primary source of income. In addition, cattle and goats are common domestic animals raised in the district, with 11% of farmers relying on them as their primary source of income. A small percentage of farmers (about 8%) earn money off the farm, primarily through petty trading, which is primarily carried out by young and female members of the community.

### 3.2 Perceived climate change and local indicators

Climate change, according to focus group participants and KII, has posed serious obstacles in the lives of farmers in West Hararghe. As a result, 88% of respondents across three study sites, including 88% in Yabdo Shembeko, 90% in Saro, and 85% in Aribu Rekete kebeles, said climate change is a pressing issue. Discussants stated that a change in the local climate has been noted in the Chiro district since 1985, which was Ethiopia's worst drought era in recent history. As a result, many people were forced to flee to other parts of the country, notably Wollega in western Ethiopia. According to respondents, climate change and variability are exemplified by low rainfall (88%), high local temperatures (89%), unpredictability in rainy periods (93%), agro-ecological shift (82%), and the frequent occurrence of drought (84%) (Table 1). Furthermore, farmers noted a decrease in soil fertility, a rise in pest frequency, a decrease in water resources, a decline in yearly harvest, and a shortage of pasture for animals as other indicators that climate change exists. The finding is in line with study conducted in south Ethiopia [67].

**Table 1:** Perceived climate change and indicators in three kebeles of West Hararaghe, Ethiopia.

Local indicators	Study *kebeles*			Average (n = 273)
	Yabdo Shembeko (n = 78)	Saro (n = 71)	Aribu Rekete (n = 124)	
	%	%	%	%
Rainfall reduction	90	90	85	88
Temperature increased	94	94	80	89
Availability of water resources reduced	82	87	76	82
Occurrence of drought increased	86	90	76	84
Unpredictability of rainfall	95	94	91	93
Agro-ecology shift	82	80	83	82
Reduction in crop yield	88	93	87	89

Farmers used to be able to predict the start and end of the rainy season, but this is becoming increasingly difficult due to its fluctuation. Farmers, for example, frequently encounter a lack of rain during the season they expect for growing crops, and the end of the rainy period also fluctuates. Furthermore, the amount of rain varies; at times it is intense, while at others it is far less than expected. In the past, it rained nonstop throughout the summer (rain season). However, in recent years, rainfall patterns have shifted, and the main rainy season now appears to be short. A study by Ref. [20] in northern Ethiopia also reported a fluctuation in rainfall period and depth, showing the onset of rainfall is unpredictable. Similarly, the temperature of the area has changed in comparison to previous years. Discussants stated that at the start of the dry season, the area's temperatures become high and poses a health risk to residents. According to one of the key informants, from the Chiro district's agriculture and natural resource office, the current weather conditions in the area are difficult, which translates to English as:

“Just after the beginning of the dry season, in October, we begin to feel very bad weather that we haven't experienced in the past. Sometimes it is very warm and other times it is cold. It could be a mixture of warm and cold weather."

According to focus group discussants and key informant interviewees, drought is another aspect of the Chiro district in the West Hararagh zone. They mentioned that drought occurred until recently, despite a decreasing trend in terms of its impact, particularly as compared to the past, e.g., 1985, but occurs more frequently now than in the past due to the cumulative effect of changes in major climate parameters such as rainfall and temperature. Droughts happened every 10 years some 10–15 years ago, but due to current climate change, notably due to a decline in rainfall, they face drought every two to five years. The FGD and KII participants attempted to recall the 1991, 2003, 2005, 2010, and 2015 droughts that severely impacted their livelihoods. Flooding caused by climate change has not been common in the area in the recent past (Table 1). That is to say, disastrous floods occur less frequently in the area than in drought events.

**Table 2:** Climate change induced impacts on the means of livelihood of West Hararaghe, Ethiopia.

Impacts on various aspect of livelihood	Yabdo Shembeqo (n = 78)	Saro (n = 71)	Aribu Rekete (n = 124)	Average (n = 273)	Rank
	%	%	%	%	
Soil fertility	77	80	84	**80**	**4**
Crop production	95	94	90	**93**	**1**
Livestock production	94	93	86	**91**	**3**
Water resources	94	94	87	**92**	**2**
Social problem	73	75	69	**72**	**6**
Adaptive capacity	85	85	72	**81**	**5**

Furthermore, pests and diseases impacting crops and livestock are another climate change-related occurrence that regularly affects farmers in the research area. Farmers encounter difficulties in precisely identifying diseases that affect their animals, yet some crop pests, such as American armyworms, have been identified as a result of their occurrence in other parts of the world. In terms of severity and frequency, the focus group discussants ranked agricultural and livestock pests and illnesses as the 4th and 5th obstacles to their livelihood. In terms of frequency, rainfall variability was ranked first, followed by temperature increased (2nd), shortage of rainfall (3rd), drought (4th), crop pest and animal disease (5th), and flood ranked (6th).

### 3.3 Perceived causes of climate change in the study site

In the study area, human actions, such as unbalanced tree cutting, are viewed as the primary cause of climate change by focus group discussants and key informants. In Ethiopia, deforestation is associated with the expansion of arable and grazing land, the use of biomass energy, and population pressure [68]. Farmers in the research area acknowledged the situation and believed they had contributed to local climate change, so they were motivated to rehabilitate the degraded land, as explained during the focus group discussion. “When we were children, individuals, including our family, created their settlement on the mountain by removing the big forest and setting the ground for farming,” farmers said during the FGD. However, they believe that, after 2004, the government began teaching us about the long-term consequences of our actions on deforestation and compelled citizens to work in watershed management. As a result, we are beginning to notice positive changes in our mountainous land today.” The devastative tree removal that led to degradation and climate change in their locality took place between 1991 and 2003 following the downfall of the Derge government in 1991. According to respondents, the biggest sources of observed weather and climate change in the area are deforestation and land degradation. In principle, even though deforestation and degradation would have an impact on the local climate, it is essential to note the global climate and hydrological cycle that could influence local situations.

### 3.4 Climate change induced impacts on livelihood

#### 3.4.1 Climate change induced impacts on crop production

Farmers in the study area believe that climate change has an impact on crop output because of its effects on soil fertility, insect, disease, and weed incidence, as well as water availability. Climate change, according to 80% of respondents, has reduced soil fertility in the area, which is the fourth most significant barrier to crop productivity and, thus, food security and livelihood (Table 2). Furthermore, farmers who participated in the discussion indicated that their crop production used to be very fruitful with rich topsoil, but that the soil has now become shallow and infertile. Climate change, including changes in rain patterns and temperature increases, has been cited as the primary reason. In the study area, farmers associated land productivity decline with climate change. Another study conducted in northern Ethiopia also concluded that climate-induced rainfall variability and shortage are the key causes of the observed land degradation and consequent food insecurity [68]. Of course, productivity is influenced by the climate, such as soil moisture and temperature and its influence on nutrient availability [69,70,71,72,73,74], but it is not the only factor that affects plant performance. For instance, continuous cultivation with insufficient soil fertility amendment and severe erosion on sloping land could gradually affect the productivity.

The majority of respondents (93%) said that climate change has harmed crop production, and they ranked it first among the consequences of climate change on the community (Table 2). The key concerns affecting crop productivity in the research area were the late and early arrival of rainfall, as well as its unpredictability within the growing season in terms of amount, distribution, and rain days. This is a challenge as farmers are not sure about the timing for preparing the seed bed. Other studies have reported the effect of climate change on crop production due to timing of the rainfall [1,75].

In addition, climate change is assumed to be the cause of many emerging weeds that affect the yield of a crop. Striga, and *Lantana camara* the major types of weeds affecting crop production in the district, and they have become more common in recent years as farmers and key informants have responded to climate change. In addition, farmers are also facing a new weed type locally known as Wonjali. By contrast, some weeds, such as Bdifesas and Bachara, used to be common in the area and severely affected the yield, which have currently disappeared from their area, perhaps due to climate change. During this study period, farmers were facing a serious challenge from pests called American armyworms, which affected only three kebeles in 2017, but after one year, it covered the whole 27 kebeles in 2018. Pests and diseases are likely to move following climate change, affecting previously immune areas and those less prepared institutionally to manage and control them, with potentially higher negative impacts [15].

Due to the extreme reduction in rainfall, farmers mentioned that the productivity of sorghum, which is the key crop of the district, was extremely reduced, challenging food security. The reduction in rainfall led to a decline in soil moisture content and a reduction in both underground and river water for irrigation. Besides, the available literature suggests that climate change will continue to impact water availability through lower and less predictable precipitation levels and higher temperatures, both of which directly affect agriculture [76]. About 92% of the respondents noted that access to water, particularly in the dry season, was among the severe impacts of climate change in the study area (Table 2).

The effect of climate change on the decline in water resources was reported by the majority of the respondents (Table 2). In the Chiro district, some ponds and rivers have dried up, while others are on the verge of drying up. According to the replies, some of the plants dry up in the winter/dry season due to the effects of climate change. As discussants and important informants pointed out, the Chiro, Beka, Gile, Medicho, and Yabdo rivers are among the district's dried waterways. Sedimentation of rivers and ponds was one of the issues mentioned. Climate change has an impact on groundwater recharge as well as the quantity and quality of water supplies [77].

#### 3.4.2 Climate change induced impacts on livestock production

Climate change impacted livestock productivity in a variety of ways in the study area, making it one of agriculture's most vulnerable sectors. Individuals and households rely on livestock for food, money, capital, draught power, and a safety net. However, livestock production is among the most severely affected sectors in the area, according to the majority of respondents (92%; Table 2). Respondents associate those problems with climate change, such as temperature increases and lack of water. Climate change may result in a lack of pasture due to a lack of rainfall and an increase in temperatures, both of which affect grass growth. Rain and temperature patterns affect cattle production systems throughout the year, affecting pasture development as well as disease and parasite outbreaks [78].

Farmers noted that “we used to feed our animals weed and crop residues like maize and sorghum,” but that “nowadays, due to dryness and low crop yield, it is not easily accessible.” Farms reported that the government provided feed for many farmers as recently as 2015, owing to a severe drought. In the past, foraging was not a major issue in the Highlands. Farmers are currently suffering additional strains on their livelihoods due to unwelcome costs associated with purchasing feed from the market. Other studies also reported that climate change could increase the prevalence of disease-causing parasites and vectors, which could have an impact on livestockand pose a major threat to many farmers who rely on livestock production [79].

#### 3.4.3 Climate change induced social problem

As discussed by the participants, disagreement and conflict among farmers as a result of resource limitations are prevalent social problems that have been occurring in the community and are impacting farmers. According to 72% of respondents, competition over water resources has been the main source of conflict in the area, particularly among individuals who own *Khat* farms (Table 2). Farmers' disputes were also said to be particularly acute when there was no rain, according to key informants. In addition, there were also disputes between farmers over pasture and grassland. In Fugnadibo kebele, for example, there was a competition to use grass from communal grazing land, which was one of the major social issues that arose after a severe event in the district and was resolved after a series of negotiations. On the other hand, forced temporary displacement in search of work wedges from rural to urban areas has produced a new social crisis by producing a labor shortage in rural areas and supporting the excessive accumulation of unemployed youth in urban areas.

About ten years ago, the impact of climate change on livelihoods created an external aid-dependent community, which is what farmers didn't experience. The decline in the productivity of soil fertility, crop and livestock production, and the subsequent reduction in farmers' income affected the ability of farmers to send their children to school and to cover health service and other social costs. In order to fill these social gaps, some segments of the farming community have begun to rely on food and other external aid, especially from CARE (Cooperative for Assistance and Relief Everywhere) Ethiopia. Furthermore, due to a decline in productivity, a scarcity of free arable land, a lack of economic sectors that can employ youth, and a reduction in the livelihood dimensions, the majority of youths migrate into the city and abroad, such as Arab countries. Such movements, particularly to and from abroad, were sometimes carried out illegally, resulting in abuse of power and loss of life in the desert A wide range of social problems are observed and perceived as somehow due to climate change in the study area. Other studies report the social impacts of climate change, including food insecurity, joblessness, and poverty [80]. Of course, climate change affecting the farming community could influence the social components, but it could not be the only factor affecting the social condition of the community as other factors such as population increase and the national economy also interplay.

#### 3.4.4 Implication of climate change induced impacts on adaptive capacity of farmers

The result shows that 81% of respondents reported that the impact of climate change on land, water resources, and economic capacity is the key impact, diminishing the adaptive capacity of rural households for an uncertain future (Table 2). Land degradation due to erosion that contributes to the removal of plant nutrients and water shortages were among the pressing challenges affecting the adaptive capacity of the area. The declining trends of water potential are a constraint that has been affecting livelihoods and is expected to continue as pressure on vulnerable livelihoods. On the other hand, the collective effect of climate change through degraded land and declining water resources affected agriculture (i.e., crop and livestock productivity), affecting the economic capacity of farmers. The decline in the natural and economic aspects of farm households is the key factor that exposes the household or society to future climate extremes, increasing the vulnerability and reducing the adaptive capacity. The adaptive capacity of a community is its willingness to take the initiative in making adjustments to mitigate the negative effects of climate change as well as the ability to respond to climate change and then initiate responses to these climate changes [81]. This could be influenced by the economic capacity of the community. At the individual or household level, the adaptive capacity is affected by individuals’ ability to perceive and understand climate risks, access to financial capital and assets, human and social capital, and access to information and technology [82]. Enhancement of adaptive capacity can reduce vulnerability and promote sustainable development across many dimensions [83]. Climate change in the study area has implications for the adaptive capacity of farmers.

### 3.5 Climate change induced livelihood shift

About 86% of respondents, focus group discussants, and key informants believe that farmers have been changing their livelihood from crop production to Khat production in the West Harareghe zone of Ethiopia. This could be associated with some factors, including income and climate change. Climate change-induced yield reductions in crop production and the consequent shrinkage of individual livelihoods were the important reasons for farmers' decision to progressively shift their livelihoods. The decline in soil moisture due to a fluctuation and long dry spells and the subsequent decline in the yearly harvest from the crop has motivated farmers to widely adopt the *Khat* plant, as focus group discussants and key informants noted. One of the outweighing advantages of *Khat* production is that it can be harvested at least twice a year under rain-fed conditions, while up to five harvests per year are possible under irrigation [84]. *Khat* can grow in a range of agro-ecological conditions and can withstand climatic extremes. Sometimes smallholder farmers are engaged in off-farm and non-farm activities to diversify their livelihood strategies, thereby improving their food security [85]. But, in West Harareghe, in order to reduce the vulnerability of their livelihood, rural subsistence farmers are shifting the source of income and livelihood.

Having a high income from the small land size and a reasonable market for Khat also encourages farmers to shift to *Khat* plantations as part of adapting to climate change. The poor tolerance of food crops like maize to underlying climate change, soil degradation, and crop pests as compared to the *Khat* plant has been another advantage that inspired farmers in Harareghe, as both FGD and key informants coined. According to Ref. [34], among the benefits of the expansion of the *Khat* plant in Ethiopia is the ability of *Khat* to be grown in degraded areas, higher income returns relative to other cereal crops, and less vulnerability to drought stress. In addition to these, increased market opportunities, favorable prices, low risk, low labor inputs, low susceptibility to weed infestation, and the prevalence of pests and diseases compared with cereal crops are the factors that triggered farmers in Habro district to change from annual crops to *Khat* plantation [31,36]. The challenge with this form of climate change adaptation is that farmers producing food crops decline. A key informant said that currently, many farmers are in the market to buy food items and sell *Khat*. The expert working at the district level expressed his worry as to “What will happen in the future if the majority of farmers go out to buy food items instead of selling food (cereal) in the market?” **Role of** ***Khat*** **in climate change adaptation and livelihood**.

*Khat* growing is widely practiced, covering more than half of the cultivated land of the West Harareghe zone. Discussants indicated that *Khat* is sold in both the local and international markets and the income from it is currently higher than that of cereal crops. Focus group discussants and key informants noted that the cost of production is very low compared to other competing enterprises due to low labor demands for growing Khat and less risk of failure due to climate variability and soil fertility decline. When compared to other crops, *Khat* gives a high income per unit of land. Therefore, the discussants indicated that the current land holding capacity is very low and that the *Khat* crop reduces its vulnerability to the impacts of climate change. Farmers who have a small farm size can produce *Khat* in an intensive manner and harvest a minimum of three times per year Thus, in West Hararghe, farmers prefer to grow *Khat* as part of climate change adaptation as well as an attempt to get a better income compared to growing cereals. According to studies conducted in various parts of Ethiopia, *Khat* production requires fewer inputs and has a more stable price in the global market than coffee, so farmers prefer to grow *Khat* [86].

The contribution of *Khat* to climate change adaptation and livelihood appears indirect and relies on the market. On the other hand, concerning the livelihood value, *Khat* is not a crop of any part that is eaten as food rather than people chewing its leaves just to gain the psycho-stimulant effect from the alkaloid chemical ingredient ‘cathinone*’* present in the fresh leaves of the plant. Regarding this, many researchers have reported that it has negative health and socio-economic effects. Among scholars [48,87],reported that, when consumed, *Khat* can cause some disorders, including sleep, appetite, depression, and stomach Thus, the role of *Khat* in climate change adaptation should not be viewed only from the perspective of income.

### 3.6 Response strategies to climate change induced impacts

Rural people used a variety of adaptation strategies, which could be classified as technological, behavioral, managerial, or policy-driven. Among the technological strategies, construction of stone and soil bands (849%), terracing (85%), using fertilizer (68%), improved seed (74%), and irrigation (77%) through water pumps were the most commonly used to adapt to the impact of climate change in West Harareghe. Planting tree seedlings (81%), which is the result of deliberate policy in combination with technological strategies such as constructing soil and water conservation structures using stone and soil bunds, terracing, and protecting the maintained area, were the major mechanisms farmers implemented in order to rehabilitate degraded lands (Table 3). Watershed management practices are strategies for dealing with and adapting to current shocks. Besides, improved seed and fertilizer were used to increase the productivity of crop production to adapt to the impacts of climate change.

**Table 3:** Climate change adaptation strategies in three *Kebeles* of West Hararaghe, Ethiopia.

Adaptation strategies	Yabdo Shembeqo (n = 78)	Saro (n = 71)	Aribu Rekete (n = 124)	Average (n = 273)
	%	%	%	%
**Technological**				**78**
Construction of stone & soil bund	86	85	82	84
Terracing	87	83	84	85
Using fertilizer	65	68	70	68
Using improved seed	68	75	80	74
Irrigation	82	77	73	77
**Behavioral**				**86**
Livelihood shift	88	87	84	86
**Managerial**				**82**
Intercropping	77	75	87	80
Drought tolerant crop varieties	82	83	86	84
Petty trade	86	90	79	85
Cut & carry system	77	83	79	80
**Policy driven**				**77**
Productive Safety Net Program (PSNP)	72	77	66	72
Planting seedling	79	86	77	81

The behavioral categories of adaptation were autonomously practiced by *Khat* production (86%), replacing crop production as a means of living with the changing climates (Table 3). This spontaneous adaptation encourages farmers to adapt, particularly to reduced rainfall. The high vulnerability of different cereal or other food crop items to either a shortage of rainfall or an increase in temperature and the relative tolerance of *Khat* plant to these extremes underpin the farmers' decision to widely adapt *Khat* plant as among the palatable options to the impacts of climate change.

In terms of managerial adaptation, feeding livestock through cut and carry (80%), intercropping (80%) maize and *Khat* plants, and using drought-resistant crop varieties (84%) such as sorghum were the most common practices that farmers used to adapt to the bleak situation (Table 4). Change from long-maturing crop varieties to short-maturing crops was the major strategy farmers applied to adapt to climate change. Previously, the existing local varieties of sorghum needed six months to mature, but the newly emerged varieties take only three months. These new varieties of sorghum are very advantageous to farmers today when the challenges of a short period of rain are becoming common. However, farmers are not happy with this new variety of sorghum because of its low forage yield for their animals as compared to the local variety. When they sow sorghum and maize, their aim is to get food forage. As a result, they are currently looking for improved varieties that are efficient in solving the challenges of both food and forage needs.

**Table 4:** Regression result for determinants of farmers’ adaptation.

Explanatory variables	Technological strategies		Behavioral strategies		Managerial strategies		Policy derive strategies	
	Marginal Effects	P-Value	Marginal Effects	P-Value	Marginal Effects	P-value	Marginal Effects	P-Value
Age	−0.290	0.116	−0.003	0.586	−0.080	0.215	0.006	0.758
Lowland agro-ecology	0.421	0.304	0.029	0.787	0.647*	**0.059**	0.251	0.551
Wealth	1.268***	**0.000**	0.023	0.631	−0.007	0.358	−0.006	0.302
Yield reduction	−0.637**	**0.015**	0.157**	**0.006**	0.351	0.403	0.613**	**0.006**
Educational status	0.118	0.530	0.087	0.185	−0.013	0.774	−0.064	0.213
Perceived onset of rain	0.138	0.567	0.176**	**0.011**	0.014	0.54	0.128	0.499
Soil infertility	0.110*	**0.064**	0.020	0.678	0.895**	**0.002**	0.338*	**0.081**
Sex	−0.168	0.386	−0.015	0.765	−0.620	0.147	0.199	0.459
Access to market	0.419	0.144	0.726	**0.106**	0.353	0.505	−0.004	0.902
Frequency of extension contact	−0.079	0.764	−0.513	0.269	0.078	0.743	0.238	0.181
Years of agricultural experience	0.295	0.167	−0.098	0.331	0.321	0.187	0.166	0.673
Access to credit	1.221***	**0.000**	0.011	0.860	0.289	0.393	−0.175	0.379
Institutional participation	−0.012	0.495	−0.163**	**0.004**	0.180	0.718	0.010	0.968
Land size	−0.607	0.114	−0.016*	**0.070**	−0.028	0.929	−0.062	0.806
Dependence ration	−0.073**	**0.034**	−0.181**	**0.005**	−0.113	0.682	0.551*	**0.043**
Water scarcity	0.262	0.219	0.076	0.174	0.062	0.822	0.003	0.988
Irrigation	0.216	0.317	0.052	0.454	−0.425	0.134	−0.805**	**0.002**
Access to early warning system	0.095*	**0.057**	0.011	0.844	**−0.999**	**0.002**	0.603	0.217

*, ** and *** Statistical significance at 10%, 5% and 1% respectively.

Non-farm strategies like petty trades (85%) that have been mainly practiced by women were among the other adaptation mechanisms farmers are using in the district (Table 3). As a result of their high involvement in the market, they were economically active and considered sources of finance in the household or community. In the district, men are more involved in the field, and women are more tangled up in selling *Khat*. Focus group discussants from Saro kebele reported that women are not only involved in *Khat* selling but also actively participate in other trades, including goat trading. They buy goats from one market and sell them in the other nearby market, where their price is relatively expensive. Accordingly, female discussants indicated that “we participate in petty trade; we buy Khat from farmers and take it to the Chiro market and other small markets around our area.” Besides, we also participate in other types of trading, like buying goats from one market and selling them to another market. For this, we mostly take credit from VESA (Village Economic and Social Association).

Poor farm households’ involvement in productive safety net programs (PSNP) (72%) to get external support both in terms of cash and kind aid to cope with the immediate extreme events and build the adaptive capacity to climate change is among the key adaptation strategies (Table 3). Additional adaptation options that rural poor farmers have used to adapt to the impact of climate change, as discussed by discussants and key informants, include: participation as labor in their nearby towns; moving into town areas in search of temporary work; getting provisions from the government through emergency support, or being involved in PSNP.

### 3.7 Determinants of farmers' adaptation strategies to climate change-induced impacts

The results in Table 4 show the variables that determine the farmers’ adaptation to climate change-induced impacts. Farmers used nearly identical adaptation strategies across the agro-ecology, so the determinants of adaptation strategies to the impacts of climate change-induced shocks were investigated at the site-wide level. Out of 18 variables, 12 were found to significantly influence the adaptation strategies of farmers to climate change-induced impacts.

We find that the average marginal effect of the adoption of managerial strategies in lowland agro-ecology is 0.647. This means that the probably of adapting to one of the managerial adaptation measures (such as intercropping, drought tolerant crop varieties, petty trade, and cut and carry systems) is on average about 64.7% higher than farmers in the highland agro-ecology. Concerning the wealth status, the average marginal effect of wealth is 1.268. This implies that a unit increase in the natural resource endowments or financial capital increases the farmers’ ability to adopt technological measures. These include the adoption of fertilizer, water pumps, improved seeds, and irrigation practice by 126.8% as compared to the poor. This study is similar to the finding by Ref. [88], who reported that wealthier farmers are more likely to use adaptation practices mainly that need financing in response to climate change than poor farmers [89]. also reported that households with more assets are more likely to adapt to perceived climate change.

We find that the average probability of yield reduction due to climatic extremes is −0.637. This suggests that a unit reduction in yield negatively and significantly affects the probability of farmers' adoption of technologies. The yield reduction due to climatic extremes could negatively affect adoption of technological inputs such as the use of fertilizer, improved seed, water pump, and installation irrigation by 63.7%. The probable reason for this is that decreases in the amount of yield harvested are directly related to the farmers' income, which in turn determines their investment ability to adopt. On the other hand, the probabilities of yield reductions were 0.157 and 0.613 for the adoption of behavioral and policy-driven strategies, respectively. The result indicates that the farmers' probability of deciding to shift their livelihood strategies from cereal to *Khat* is 15.7% due to yield reduction. In the same fashion, the yearly yield reduction caused by climate variability extremes would increase the probability of farmers’ instability to plant seedlings and expose the farmers to external support through the productive safety net program (PSNP) by 61.3%. In the study area, those farmers who are severely affected by climatic shocks and the poorest of the poor are the ones who participate in the PSNP to fill the food gaps in their households.

The result shows that the marginal effect of a behavioral adaptation is 0.176 to the perceived onset of rainfall. This suggests that the farmers' probability to take a behavioral adaptation option increases with variability in the onset of rainfall by 17.6%. The result suggests that the change in the onset period of the rainfall is among the significant factors that influence the farmers' shifting livelihood systems. Similarly, the result indicates that the likelihood of adopting technological, managerial, and policy-driven strategies for soil infertility is 0.11, 0.895, and 0.338, respectively. This shows that farmers’ probability to adopt diversities of options under technological, managerial, and policy measures in order to adapt to the impacts of soil infertility that are particularly caused by rainfall variability and erosion is 11%, 89.5%, and 33.8%, respectively.

The marginal effect of access to the markets is 0.726 for the probability of adopting behavioral strategies. A unit increase in access to the market, particularly for selling *Khat* and buying technologies (water pump), increased the farmers' ability to decide to adopt behavioral variables or increased the probability of farmers’ to shifting their livelihood from intensive cereal production practice to *Khat* crop plantation. The result is in agreement with a study by Refs. [90,91]. They revealed that access to the market has the potential to increase the likelihood of changing the input use intensity as an adaptive measure. Moreover, in rural areas, the market is not only the means of channel for buying and selling different commodities of production, but it is also the place where farmers get up-to-date information related to the weather and other issues associated with their livelihood. The same finding by Ref. [92] indicated that the market is an important determinant for the adaptation method because, apart from using the channel, it serves as a means of exchanging information with other farmers, including about the current trends of climate and their livelihood challenges, and thereby enable them to adopt other recommended means of livelihood.

The survey results revealed that the average probability of accessing to credit is 1.221 for adopting technological strategies to mitigate the impacts of climate change and variability-induced shocks. A household that has access to getting credit from any financial institution is assumed to increase the probability of adopting technological measures by 122.1% more than its counter parts. This is because the adaptation of technologies such as fertilizer, improved seeds, water pumps, and others needs finance to invest. In the same fashion [93], Pickson and He (2021) stated that economic resources such as credit services, government subsidies, diversity of income sources, and remittances received were all seen to increase the adaptive capacity of farmers to the impacts of climate change. In addition to this, the results of this study are in line with the findings [94,95,96].

We find that the average marginal effect of adopting behavioral measures is −0.163 for the farmers who have institutional participation. This suggests that farmers who have high involvement and access to institutional services like meetings and training decrease the probability of adopting behavioral strategies by 16.3%. This is because the district-level government (Agriculture and Natural Resources Office) and non-government institutions (CARE in Harareghe) that we interviewed during the fieldwork were not happy with the progressive shifting of the farmers' livelihoods. They aggressively mention that “although the *Khat* crop is more resistant to the current climatic extremes as compared to foodstuff crops, it is a must to strive a lot to adopt different strategies to adapt to the underlying climate variability. Because fully being dependent on the market even for consumption, is very dangerous. For instant, what if other communities are also affected by severe extremes like drought and if they themselves the challenge of feeding themselves and don't offer any food items to the market”, noted three experienced experts in the agricultural and natural resource offices.

The result indicates that the likelihood of an adapting behavioral strategy due to a decline in the land size is 0.016. A unit decline in the land size negatively and significantly decreased the adaptation ability of farmers by 1.6%in the face of climate change-induced shocks. This indicates that the smaller the size of the farmland, the greater the probability of farmers shifting in to *Khat* plantations from cereal crop production. Because one of the possible growing pull factors for *Khat* production instead of cereal crops is its small land requirement, its ability to be harvested at a regular interval (up to six harvests per annum), and that income derived from *Khat* could enable farmers to rent additional farm lots for seasonal cropping [31]. Furthermore, *Khat* is grown on plots that are unsuitable for other crops, and cereal crop production land size cannot be decreased [41].

The result of the average marginal effects of the probability of adopting technological, behavioral, and policy-driven strategies is −0.073, −0.181, and 0.551 for the dependency ratio, respectively. The addition of a single dependent individual to the vulnerable farm household increases the challenge of adopting technological farm inputs and behavioral measures by 7.3% and 18.1%, respectively. This is because farmers face an additional challenge in feeding the increased number of households, which affects their financial capacity to invest in production-improving farm technologies. On the other hand, the increase in the number of dependent family sizes increased the participation of poor farmers in PSNP activities to cope with the climatic shocks by 55.1%. Because the productive safety net program is the strategy that is managed by the government and development practitioners in collaboration with the aim of reducing hongs in one hand and increasing the productive capacity of the area through soil and water conservation practice. This means the increase in the dependent family size means it increases the vulnerability of the households to climatic shocks. Contrary to the present finding, the likelihood of adaptation to climate change was higher with a large household size than with a small household size [97]. This implies that an important number of families are active participants in the production system for the well-being of the household. In this situation, having a large family size means households have a better chance to allocate their own labor for the most important activities of multiple cropping, which is the field operation [98].

The marginal effect of irrigation is −0.805 for adopting policy-driven strategies. This suggests that the probability of adopting policy-driven strategies like receiving external support by involved in PSNP decreases by 80.5% for farmers’ who are involved in small-scale irrigation. From the very beginning, the farmers who involved in irrigation practices are the better-off because they required water pumps and other intensive farming technologies, and most probably they were *Khat* producers. That means they have a better income and do not face the challenge of feeding themselves, as poor farmers do, and they do not look for external support [99]. stated that the irrigation experience of farmers is becoming an important adaptation tool to increase agricultural production. In the present study area, this is the measure that treated the income that was severely affected by the impact of climate change-induced shocks.

Another important strategy that is likely to increase the adaptation ability of farmers to the impacts of climate change-induced shocks is increasing access to early warning systems. The marginal effects of farmers’ probability to adopt technological and managerial strategies are 0.095 and −0.999 for farmers who have better access to early warning systems. From this, we can conclude that the probability of farmers adopting different technological inputs would increase by 9.5% among those farmers who have no access, to early warning systems as compared to those who have no access keeping all other factors constant. On the other hand, farmers' ability to adapt managerial strategies such as land preparation, cultivation, intercropping, investment to adopt drought-tolerant crop varieties and others would decrease with limited early warning communication by 99.9%. Therefore, the government should provide pertinent attention to establish an effective working early warning system that can support local area-specific strategies and help smallholder farmers live with the changing climate and its induced extreme events [100]. Because in Ethiopia, early-informing farmers about the likely variability of climatic elements significantly increases the intensity of their adaptation ability to the impacts of climate change [101].

## 4 Conclusion and recommendation

The impacts of climate change, in conjunction with the progressive shift of smallholder livelihoods, were investigated. Changes in major climatic elements such as temperature and rainfall were the most significant direct effects of climate change. Droughts, the prevalence of pests and diseases, floods, and the shifting trend of agro-ecological conditions were the notable indirect impacts of climate change that were perceived in the local area. The reduction in soil fertility and the unpredictability of the onset and distribution of seasonal rainfall, coupled with the prevalence of pests and weed infestations, were the key causes of the low crop production. The prevalence of livestock diseases, forage scarcity, and water shortages were challenges in livestock production.

To deal with the current situation of climate change and food insecurity, smallholder farmers are gradually shifting their livelihoods from cereals to *Khat* plantations. Of the respondents and discussants, they considered *Khat* as the most viable means of livelihood strategy in the face of current climatic conditions in the area due to its ability to tolerate water shortages, little irrigation water demand, tolerance to drought, low risk of failure, and low susceptibility to weed and disease infestation. Growing *Khat* has other advantages, such as low labor demand, a large market, higher income than cereals, and a high yield from small-scale land. In order to increase the adaptive capacity and reduce the susceptibility of rural livelihoods, farmers have been employing technological, behavioral, managerial, and policy-driven strategies. However, the current impact of climate change on livelihoods needs highly coordinated, efficient, and effective efforts of natural, material, financial, and human resources. In addition to this, yield reduction, the perceived onset of rain, soil infertility, and land size were determinant factors found to negatively and significantly impact the adaptation capacity of farmers in the study area. On the other hand, wealth, access to markets, institutional participation, irrigation, and access to early warning systems were found to positively and significantly influence the adoption decisions of adaptation strategies by farmers in the West Haraghe zone.

Therefore, governments and non-government organizations are advised to implement activities that can increase the productivity of natural resources such as land and water. That can be realized through exciting both biological and structural soil and water conservation activities (e.g., tree planting, constructing soil and stone bands, tracing, and mulching), which would help to improve the productivity of farmland and increase the water reservoir to have better access, i.e., for domestic use, livestock, and irrigation practices. In addition to this, we suggest that the farmers’ tendency to shift from sowing of food crops into stimulant crop types is not a reliable system of livelihood. Because being dependent on the market for the basic needs of consumption, in the long run, will not be the safest path. Furthermore, under uncertain climatic conditions, the situation could be worst perhaps what if production in other corner of the country on which they are assuming to rely is hardly affected by climatic extremes. Therefore, the researchers interested to forward particular recommendation for both farmers and local planners to reconsider the risk or profitability of their new way of life and switch to food crops instead of *Khat*, assuring food security and a long-term existence.

## Funding

The research fund for this study was obtained from the 10.13039/100000200United States of America for International Development (10.13039/100000200USAID) under the Productive Safety Net Programme (PSNP).

## Data availability

The first author will provide data upon reasonable request.

## Authors’ contributions

Daniel Assefa Tofu (Ass. Prof.) conceived and designed the experiments, performed the experiments; analyzed and interpreted the data; and wrote the paper. Kebede Wolka (Ph.D.) contributed reagents, materials, analysis tools, or data; and contributed by interpretation of the results, and write-up of the paper. The authors read and approved the final manuscript.

## Declaration of competing interest

We declare that there is no known conflict of interest in this research output.
